# Post-surgical surveillance of locally advanced ileal carcinoids found by routine ileal intubation during screening colonoscopy: a case series

**DOI:** 10.1186/1752-1947-8-444

**Published:** 2014-12-19

**Authors:** Eileen M Ten Cate, Leslie A Wong, Walter L Groff, Aaron T Miller

**Affiliations:** 51 Department of Family and Community Medicine, University of Toronto, 500 University Avenue, Toronto, ON M5G 1V7 Canada; 52 Department of Surgery, Columbia University, MHB-7GS-313, 177 Fort Washington Avenue, New York, NY 10032 USA; 53 Department of Surgery, Overlook Medical Center, 33 Overlook Road, Suite 412, Summit, NJ 07901 USA

**Keywords:** Carcinoid, Colonoscopy, Ileal carcinoid, Ileal intubation, Ileocolectomy, Small bowel

## Abstract

**Introduction:**

Carcinoid tumors are the most common type of small bowel tumor, and the incidence is rising. The majority of small bowel carcinoid tumors arise within 60cm of the ileocecal valve. The addition of ileoscopy to screening colonoscopy can detect asymptomatic small bowel carcinoid tumors and improve long-term prognosis through early surgical resection. Ileoscopy is a brief procedure with a high success rate and minimal complications beyond those of colonoscopy. The use of ileoscopy during screening colonoscopy has led to an increase in the early-stage detection of locoregional small bowel carcinoid tumors that can be completely treated with surgery alone, and as such has improved long-term prognosis in these patients.

**Case presentations:**

Five asymptomatic Caucasian patients, 3 males and 2 females, from 53 to 70 years old (mean age, 60 years old), were diagnosed with locoregional ileal carcinoids during routine colonoscopy with ileoscopy. Since having an ileocolectomy and without adjuvant treatment, no patient has developed tumor recurrence over a follow-up period of one and half to 12 and a half years.

**Conclusion:**

The early detection of carcinoid tumors by ileoscopy during screening colonoscopy can lead to increased long-term survival in patients with locally invasive disease. The high success rate and brief duration of the procedure, in addition to the lack of associated morbidity and mortality suggest that with further studies, routine ileoscopy during colonoscopy may be promising in the diagnosis of small bowel carcinoid tumors.

## Introduction

Carcinoid tumor is the most common tumor of the small bowel, and its incidence is rising [1]. Patients with small bowel carcinoid tumors (SBCTs) are typically asymptomatic until they reach an advanced stage, when they commonly present with non-specific abdominal pain, intermittent small bowel obstruction and/or carcinoid syndrome [2]. Because the majority of SBCTs are found in the distal 60cm of the ileum, a significant portion can be visualized by ileoscopy [1]. The addition of ileoscopy to routine screening can detect early asymptomatic SBCTs and improve long-term prognosis through early intervention [3]. Patients with symptomatic SBCTs typically present between the ages of 60 and 70 years, a population that already undergoes routine colonoscopy in the United States to screen for colon cancer (offered from age 50 onward) [1]. When SBCTs are caught at the early locoregional stage involving local tissues and lymph nodes, ileocolectomy can be curative without the need for the extensive resection and adjuvant therapy required in more advanced distantly metastatic disease [4].

Recent case series by Yarze *et al.* [5] and Lee *et al.* [6] have shown promising results in the use of screening ileoscopy for the diagnosis of asymptomatic SBCTs that suggest a decrease in morbidity and mortality, albeit without post-surgical follow-up. The aim of this report is to show that ileoscopy during screening colonoscopy can improve morbidity and mortality associated with SBCTs on a long-term basis, and to argue for the addition of ileoscopy to the practice of screening colonoscopies.

## Case presentation

We document the cases of five patients aged 53 to 70 years old (mean age, 60 years old) diagnosed with SBCTs during screening colonoscopy with ileoscopy. All patients demonstrated localized lymph node metastases on ileocolectomy, but no distant metastases by computed tomography. No patient received adjuvant therapy. To date, no patient has experienced a recurrence of the carcinoid tumor or metastatic disease.

### Case 1

A 65-year-old asymptomatic Caucasian man presented for screening colonoscopy with ileoscopy that revealed a terminal ileum lesion, which proved to be a SBCT. He underwent an open ileocolectomy, which revealed a 2.7 cm by 2.0cm malignant carcinoid tumor with non-contiguous mesenteric metastasis. No lymphadenopathy greater than 1 cm was found, however four out of ten mesenteric lymph nodes were positive for metastases. His cecum and Gerota’s fascia were also involved in the tumor and removed. On long-term follow-up, our patient remains asymptomatic with no evidence of disease 12.5 years after surgery.

### Case 2

A 53-year-old asymptomatic Caucasian man was diagnosed with SBCTs during screening colonoscopy with ileoscopy that revealed a protuberant ileocecal valve mass. He underwent an open ileocolectomy that revealed a 2.5 cm by 2.2 cm ulcerated carcinoid tumor of the distal ileum prolapsing through the ileocecal valve, with four mesenteric lymph nodes positive for metastases. On long-term follow-up, our patient remains asymptomatic and free of disease 11 years after surgery.

### Case 3

A 50-year-old asymptomatic Caucasian woman presented for screening colonoscopy because of a family history of colon cancer. A submucosal tumor of greater than 2 cm was noted in her ileum, found to be a carcinoid tumor. An open ileocolectomy revealed a 0.7cm carcinoid tumor with submucosal involvement and three out twenty four lymph nodes positive for metastases. On long-term follow-up, our patient remains asymptomatic 11 years after surgery.

### Case 4

A 65-year-old asymptomatic Caucasian man presented for screening colonoscopy with ileoscopy that revealed a SBCT in his terminal ileum. He underwent an open ileocolectomy, which demonstrated a 2 cm SBCT of the ileum with three out of thirty eight mesenteric lymph nodes positive for metastases. Our patient demonstrated no evidence of carcinoid tumor disease until his death 7 years after surgery, from small cell lung cancer.

### Case 5

A 62-year-old asymptomatic Caucasian woman presented for screening colonoscopy with ileoscopy. The ileoscopy revealed a biopsy-proven ileal carcinoid tumor (8mm), which was partially resected by endoscopy. A laparoscopic ileocolectomy was performed to completely excise the tumor, and revealed a 3.8 mm by 2 mm SBCT with tumor invasion into the submucosa. five out of fourteen mesenteric lymph nodes were positive for metastases. On follow-up our patient remains asymptomatic one and a half years after surgery.

## Discussion

Our case series supports routine ileal intubation at the time of screening colonoscopy to facilitate early diagnosis of SBCTs involving the terminal ileum, which ultimately will improve long-term prognosis. Our five patients diagnosed with regional ileal carcinoids all underwent successful surgical treatment as the sole therapy. All patients showed no evidence of disease one and a half to twelve and a half years after surgery, with one patient who died of an unrelated malignancy.

As SBCTs spread undetected, patient prognosis worsens. Landerholm *et al.* found that as small bowel carcinoids spread from local to regional to distant metastases, the five-year survival rates decrease from 100% to 86% to 51% respectively [3]. The overall survival of patients with regional lymph node involvement decreases by 14% compared with that of patients with solitary tumors [3], thus our patients with regional lymph node involvement at the time of surgery did not have a markedly worse prognosis beyond that of a solitary lesion. However with development of undetected later stage disease, the five-year survival would have decreased by nearly 50%, suggesting that the longevity of patients with asymptomatic SBCTs can be markedly improved by screening ileoscopy.

Because the diagnostic yield of ileoscopy in asymptomatic patients undergoing screening colonoscopy is low, some authors argue against its routine use [7]. These detractors generally agree that ileoscopy in screening colonoscopy should be used in selected symptomatic patients or for training purposes, but that the diagnostic yield is too low in asymptomatic patients to warrant the additional time and effort on a regular basis. It is true that the diagnostic yield of ileoscopy during screening colonoscopy is a relatively low 2.0% to 7.2% [8]. However as SBCTs increase in incidence, the diagnostic yield of this procedure will correspondingly rise. Furthermore ileoscopy is successful in most patients (72% to 97%), has minimal complications beyond those of colonoscopy, and takes only a few additional procedural minutes to perform [5]. Early detection with routine ileoscopy can lead to early diagnosis, prompt surgical resection, and improve long-term patient outcomes without the need for adjunctive therapy.

Similar previously mentioned case series by Yarze *et al.* [5] and Lee *et al.* [6] also support the use of routine ileoscopy during screening colonoscopy to discover SBCTs, albeit without follow-up beyond the initial surgery and related pathology. In these case studies, the diagnosis of asymptomatic ileal SBCTs was facilitated by screening ileoscopy in a total of six patients. Following diagnosis, all patients in these series underwent surgical resection. Despite a lack of post-surgical surveillance in these reports, both articles drew similar conclusions supporting the usefulness of ileoscopy during routine colonoscopy. Long-term, symptom-free follow-up in our case series, ranging from one and a half to twelve and a half years, supports the idea that early diagnosis as a result of ileoscopy during screening colonoscopy can improve overall long-term survival in accordance with previous reports.

## Conclusion

Early detection through screening colonoscopy with ileoscopy, and subsequent early surgical intervention of SBCTs in this case series likely contributed to improved long-term morbidity and mortality. Given the increasing incidence of SBCTs in populations undergoing screening colonoscopy, and the high success rate with minimal increase in procedural time or complication rate, ileoscopy shows promise as a worthwhile addition to standard screening colonoscopy practice. While further research is warranted, the post-surgical asymptomatic survival seen in the patients of this case series suggests that there may be long-term benefits to routine ileoscopy during screening colonoscopy.

## Consent

Written informed consent was obtained from the patients, and for the next of kin in case 4, for publication of this case series and any accompanying images. Copies of the written consents are available for review by the Editor-in-Chief of this journal.

## Abbreviations

SBCT: small bowel carcinoid tumor.
